# BARRIERS TO CARE FOR OLDER ADULTS IN MEDICATION-ASSISTED TREATMENT FOR OPIOID USE DISORDER

**DOI:** 10.1093/geroni/igz038.509

**Published:** 2019-11-08

**Authors:** Alexis A Bender, Maggi N Robert, Nathan S Quan, Emma M Klein, Molly M Perkins

**Affiliations:** 1 Emory University, Atlanta, Georgia, United States; 2 Emory University School of Medicine, Atlanta, Georgia, United States

## Abstract

Over the last decade, the number of older adults (people over the age of 50) who misuse opioids doubled and continues to increase. People over the age of 50 also represent one of the fastest growing groups entering into and sustaining medication assisted treatment (MAT) (i.e., methadone and buprenorphine) for opioid use disorder (OUD). Despite increasing awareness of this growing at-risk population, significant knowledge gaps regarding their support and care needs persist. To begin to address these gaps, we conducted interviews with 20 treatment staff, focus groups with 18 patients and surveys with 100 patients over the age of 50 at eight diverse Opioid Treatment Programs (OTPs) participating in a 1-year pilot study (Bender, PI) funded by the Georgia Clinical and Translation Science Alliance supported by the National Center Advancing Translational Sciences. Patients in this study do not always disclose their use of MAT to non-OTP providers. When they do, participants reported numerous negative experiences with non-OTP providers, including perceived discrimination, stigma, and misunderstanding by providers about MAT. These negative experiences potentially contribute to an over reliance on OTP providers to manage age-related health conditions (e.g., COPD, hypertension). Providers report minimal training about aging and varied levels of confidence to manage these conditions. We present the experiences of patients and providers with suggestions for improving care coordination. We conclude with recommendations to improve communication among providers working with older adults in recovery from OUD.

